# A novel sequence-based predictor for identifying and characterizing thermophilic proteins using estimated propensity scores of dipeptides

**DOI:** 10.1038/s41598-021-03293-w

**Published:** 2021-12-10

**Authors:** Phasit Charoenkwan, Warot Chotpatiwetchkul, Vannajan Sanghiran Lee, Chanin Nantasenamat, Watshara Shoombuatong

**Affiliations:** 1 Modern Management and Information Technology, College of Arts, Media and Technology, Chiang Mai University, Chiang Mai, 50200 Thailand; 2 Applied Computational Chemistry Research Unit, Department of Chemistry, School of Science, King Mongkut’s Institute of Technology Ladkrabang, Bangkok, 10520 Thailand; 3 Department of Chemistry, Centre of Theoretical and Computational Physics, Faculty of Science, University of Malaya, 50603 Kuala Lumpur, Malaysia; 4 Center of Data Mining and Biomedical Informatics, Faculty of Medical Technology, Mahidol University, Bangkok, 10700 Thailand

**Keywords:** Computational biology and bioinformatics, Computational models, Machine learning

## Abstract

Owing to their ability to maintain a thermodynamically stable fold at extremely high temperatures, thermophilic proteins (TTPs) play a critical role in basic research and a variety of applications in the food industry. As a result, the development of computation models for rapidly and accurately identifying novel TTPs from a large number of uncharacterized protein sequences is desirable. In spite of existing computational models that have already been developed for characterizing thermophilic proteins, their performance and interpretability remain unsatisfactory. We present a novel sequence-based thermophilic protein predictor, termed SCMTPP, for improving model predictability and interpretability. First, an up-to-date and high-quality dataset consisting of 1853 TPPs and 3233 non-TPPs was compiled from published literature. Second, the SCMTPP predictor was created by combining the scoring card method (SCM) with estimated propensity scores of *g*-gap dipeptides. Benchmarking experiments revealed that SCMTPP had a cross-validation accuracy of 0.883, which was comparable to that of a support vector machine-based predictor (0.906–0.910) and 2–17% higher than that of commonly used machine learning models. Furthermore, SCMTPP outperformed the state-of-the-art approach (ThermoPred) on the independent test dataset, with accuracy and MCC of 0.865 and 0.731, respectively. Finally, the SCMTPP-derived propensity scores were used to elucidate the critical physicochemical properties for protein thermostability enhancement. In terms of interpretability and generalizability, comparative results showed that SCMTPP was effective for identifying and characterizing TPPs. We had implemented the proposed predictor as a user-friendly online web server at http://pmlabstack.pythonanywhere.com/SCMTPP in order to allow easy access to the model. SCMTPP is expected to be a powerful tool for facilitating community-wide efforts to identify TPPs on a large scale and guiding experimental characterization of TPPs.

## Introduction

Proteins are one of the most important biological macromolecules as they perform a variety of functions such as enzyme catalysis, ion and molecular transport, antibody production, and cellular/physiological activity regulation. Protein activities are heavily influenced by the three-dimensional structure of the protein^1^. Furthermore, protein and protein complex structures provide a wealth of information for understanding inter-residue interactions such as protein folding mechanisms, folding and unfolding rates, protein structure stability, stability upon mutation, recognition mechanisms of protein–protein, protein-nucleic acid, protein–ligand complexes, which are instrumental for structure-based drug design^2,3^. Thermophilic proteins (TPPs) have already been established a critical role in biotechnology and chemical processing^4^. TPPs are stable at high temperatures of about 80–100 °C and environmental temperature of the host organism^5,6^. Additionally, specific amino acid properties such as shape, Gibbs free energy change of hydration in native proteins, dipeptide composition, contacts between amino acid residues, number of ion pairs, hydrogen bonds, packing, and aromatic clusters all play an important role in TPP stability^5,7^. According to a thorough examination of all interactions, hydrophobicity is the most important feature in TPP stability, followed by ion pairs and hydrogen bonds^8^. Understanding the molecular basis of protein thermostability is critical for designing proteins for specific industrial and medical applications that necessitate special stability^3^. Furthermore, TPPs are resistant to denaturation by chemical compounds such as detergents, surfactants, oxidizing agents, and proteases^9,10^. As a result of these properties, TPPs can be easily purified by heat treatment and can withstand harsh industrial conditions for a longer period of time^11^. It should be noted that higher thermostability of therapeutic proteins can extend their blood survival time^12^. As for their advantages in high-temperature industrial catalysis, TPPs have reduced contamination, easy mixing with low viscosity and high mass transfer rate, higher solubility of substrates and products^13^. Furthermore, the advantage of TPPs are their use in high-temperature pelleting process^14^ and in endothermic processes such as the isomerization of glucose to generate high fructose syrups^15^. Although experimental methods are the way to certify thermostability of proteins, these methods are usually labor-intensive, time-consuming and expensive. Thus, it is desirable to develop a rapid and accurate approach for identifying TPPs from a large collection of proteins.

Several previous studies have shown that machine learning (ML)-based tools can accurately characterize various protein functions using only protein primary sequences^16–24^. Several computational efforts based on machine learning (ML) methods have been made in recent years to identify TPPs^20,21,24–33^ as summarized in Table 1. As can be seen from Table 1, support vector machine (SVM) method is the most widely used technique for identifying TPPs^20,21,24–26,28–30^. For instance, Zhang and Fan^31^ developed the first TPP predictor based on amino acid composition (AAC) descriptors. Particularly, they developed a TPP predictor using the partial least squares (PLS) method on a small set of training data (76 TPPs and 76 MPPs). Afterwards, the same group^32^ introduced a LogitBoost predictor based on a larger number of data consisting of 3521 TPPs and 4895 MPPs (called *Zhang2007*). In 2008, Gromiha et al.^27^ established a new dataset (called *Gromiha2008*) by applying the CD-HIT program^34^ using a threshold of 0.4 on the *Zhang2007* data so as to remove additional redundant sequences. In 2011, Lin et al.^20^ constructed a more reliable benchmark dataset containing 915 TPPs and 793 non-TPPs (called *Lin2011*). Using this dataset, ThermoPred was developed by means of the SVM method in conjunction with AAC and dipeptide composition (DPC), which could achieve an improvement in accuracy (ACC) of 0.933 as evaluated by the jackknife cross-validation in their comparative analysis with the model of Gromiha et al.^27^. In addition, Fan et al.^25^ introduced a new TPP predictor (called PSSM400_pKa) based on the SVM method and trained on three different feature encodings namely AAC, acid dissociation constant (pKa) and position-specific scoring matrices (PSSM). The PSSM400_pKa predictor was developed based on the *Gromiha2008* dataset and its predictive performance was validated by using two independent test datasets where the *Gromiha2008* data and two independent test datasets are referred to as *Fan2016*.

**Table 1:** Summary of existing ML-based models for thermophilic protein prediction.

Author (year)	Classifier ^a^	Features ^b^	Evaluation strategy^c^	Web server availability^d^
Zhang et al.^31^	PLS	AAC	5CV/IND	No
Zhang et al.^32^	LogitBoost	AAC	5CV/IND	No
Gromiha et al.^27^	NN	AAC	5CV/IND	No
Montanucci et al.^21^	SVM	AAC, DPC	5CV	Not accessible
Lin et al.^20^	SVM	AAC, GGAC	Jackknife	Yes
Wang et al.^24^	SVM	AAC, DPC, PCP, CTD	5CV	No
Nakariyakul et al.^28^	SVM	AAC, DPC	5CV/IND	No
Zuo et al.^33^	KNN	AAC	Jackknife	Not accessible
Wang et al.^30^	SVM	AAC, GGAC	5CV/IND	No
Fan et al.^25^	SVM	AAC, pka, PSSM	10CV/IND	No
Tang et al.^29^	SVM	k-mer	5CV	No
Feng et al.^26^	SVM	ACC, DPC, PCP,RAAC	10CV/IND	No
Charoenkwan et al. (this study)	SCM	DPS	10CV/IND	Yes

^a^*KNN* k-nearest neighbor, *NN* neural networks, *PLS* partial least-square regression, *SVM* support vector machine.

^b^*AAC* amino acid composition, *CTD* composition-transition-distribution, *DPC* dipeptide composition, *DPS* dipeptide propensity scores, *GGAP* g-gap dipeptide composition, *k-mer* fragment-based technique, *pka* acid dissociation constant, *PCP* physicochemical properties, *PseACC* pseudo amino acid composition, *PSSM* position specific scoring matrix, *RACC* reduce amino acid composition, *TC* tripeptide composition.

^c^*5CV* fivefold cross-validation, *10CV* tenfold cross-validation, *jackknif* jackknife cross-validation, *IND* independent test.

^d^Not accessible: the webserver was not functional during the preparation of this manuscript.

Although existing methods could achieve good predictive performance, their overall utility is limited in terms of interpretability and practical utility. The following important issues are needed to be addressed. Firstly, SVM-based predictors are not easy-to-use and difficult for biologists and biochemists to implement on their own datasets. On the other hand, the ability of biologists and biochemists in understanding the resulting model is of great importance if they are to be applied in a real-world setting. Secondly, existing datasets do not include comprehensive TPPs and non-TPPs. Therefore, these datasets might not have sufficient information necessary for the development of comprehensive TPP predictors. Finally, almost all existing methods (with the exception for ThermoPred^20^) did not provide a web server for public usage therefore their practical application is quite limited.

**Figure 1:**
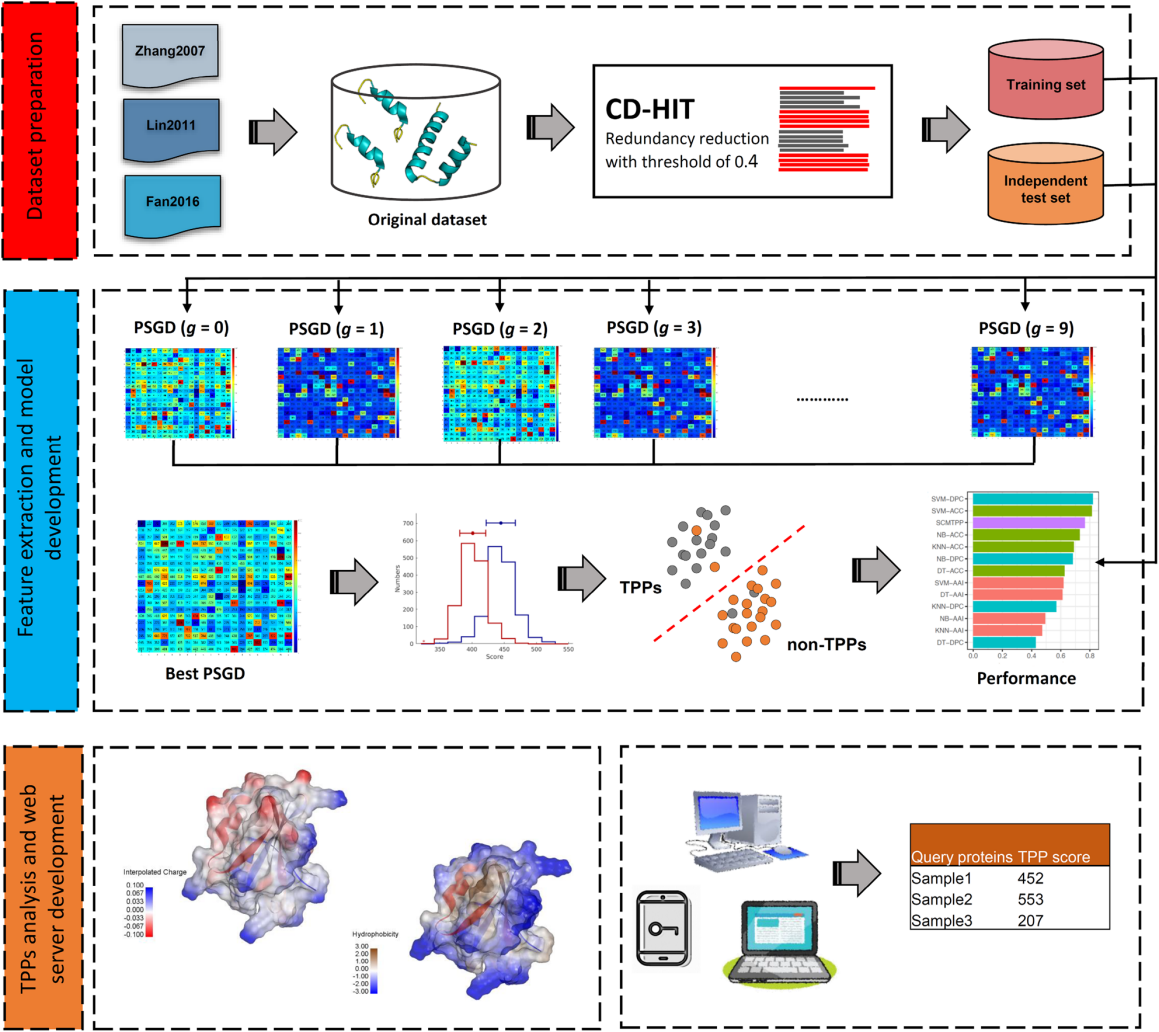
Schematic framework of the development of SCMTPP. This can be summarized into five main steps: (i) Training and independent test datasets preparation, (ii) Feature extraction, (iii) SCM-based model development, (iv) TPPs characterization and (v) SCMTPP webserver construction.

In this paper, we present SCMTPP, a novel, simple-to-implement, and interpretable computational model that is designed to improve predictive performance and model interpretability for the identification of TPPs. Figure 1 summarizes the SCMTPP's overall framework. Firstly, we established an up-to-date dataset (i.e. 1823 TPPs and 3124 non-TPPs) by combining positive and negative samples from datasets of previous studies^20,25,32,35^. Secondly, propensity scores of 20 amino acids and 400 g-gap dipeptides were estimated via the scoring card method (SCM). Finally, derived propensity scores were used for the development of a prediction model (SCMTPP) based on a scoring function for determining important biophysical and biochemical properties for TPPs. Results indicated that SCMTPP could outperform existing methods and widely used ML-based classifiers in terms of simplicity, interpretability, and practical application (according to tenfold cross-validation and independent tests).

## Materials and methods

### Dataset preparation

In this study, we created an up-to-date dataset by combining previously reported datasets consisting of *Zhang2007*^32,35^, *Lin2011*^20^ and *Fan2016*^25^. Particularly, *Zhang2007*^32,35^, *Lin2011*^20^ and *Fan2016*^25^ datasets contained 8419, 1708 and 4684 sequences, respectively. Herein, these TPPs and non-TPPs were considered as positive and negative samples, respectively. Particularly, the positive dataset was extracted from thermophilic organisms^20,25,31,32^ while the negative dataset represents the integration of non-TPPs and mesophilic proteins (MPPs) extracted from non-thermophilic organisms (i.e. *Lin2011*^20^) and mesophilic organisms (i.e. *Zhang2007*^32,35^ and *Fan2016*^25^), respectively. From these, we excluded protein sequences containing nonstandard letters such as “B”, “U”, “X”, or “Z”. Subsequently, redundant sequences were removed by applying the CD-HIT program using a threshold of 0.4 on both positive and negative datasets so as to avoid overestimation of the model performance. As a result, a total of 4945 sequences containing 1823 TPPs and 3124 non-TPPs were obtained and considered as the largest and up-to-date dataset in this aspect. Among these, we randomly selected 80% of the positive dataset containing 1482 TPPs and an equal number of non-TPPs from the negative dataset to construct a training dataset called TPP-TRN (1482 TPPs and 1482 non-TPPs). In the meanwhile, the remaining set of TPPs and an equal number of non-TPPs were considered as the independent test dataset called TPP-IND (371 TPPs and 371 non-TPPs). For reproducibility purposes, the TPP-TRN and TPP-IND datasets can be downloaded from our web server (at http://pmlabstack.pythonanywhere.com/SCMTPP).

### Feature representation

The *g*-gap dipeptide composition (GDC) descriptor is another variation of the DPC descriptor ($\mathrm{g}=0$) by representing the fraction of any two interval amino acids ${(\mathrm{aa}}_{\mathrm{i}},{\mathrm{aa}}_{\mathrm{j}};j-i>1)$ in a given peptide **P**. This descriptor can be formulated as:

1 $$\mathrm{GDC }\left(\mathrm{g}\right)=\left[{f}_{1}^{g}, {f}_{2}^{g},\dots {f}_{400}^{g}\right]$$

where ${f}_{i}^{g}$ is the percentage of the composition of the *i*^*th*^ ($i=\mathrm{1,2},\dots ,400$) *g-*gap dipeptide.

2 $${f}_{i}^{g}=\frac{{n}_{i}^{g}}{{\sum }_{i=1}^{400}{n}_{i}^{g}}$$

where ${n}_{i}^{g}$ represents the total number of *i*^*th*^* g-*gap dipeptide in a given peptide **P**. The dimension of the GDC descriptor is 400.

### Scoring card method

The SCM method has been demonstrated to perform admirably in terms of conceptual simplicity, ease of implementation and interpretability^16,18,36–39^. In 2012, Huang et al.^19^ firstly introduced the original SCM method. More recently, Charoenkwan et al. had developed an improved version that is designed for predicting and characterizing anticancer peptides^38^. It is well-recognized that the SCM method is effective for identifying proteins and providing information on the underlying molecular mechanism of proteins. The following points summarize the benefits of the SCM method. To begin, unlike well-known ML methods (such as SVM and NB methods), the SCM method uses only one threshold value to distinguish positives from negatives. Second, the SCM method is the most cost-effective method for performing a genome-wide prediction of any protein family. Finally, the information from the propensity scores of 20 amino acids and 400 dipeptides helps wet-lab researchers gain insights into the properties of proteins. The following describe the concepts and optimization procedures of an SCM classifier trained with GDC (g = 0):

**Phase 1***:* Preparing the TPP-TRN and TPP-IND datasets for SCM classifier development and evaluation.

**Phase 2***:* Calculating initial propensity scores of GDC ($\mathrm{g}=0$) using a statistical approach. For convenience of discussion, we denote propensity scores of the *g*-gap dipeptide term as PSGD (*g* = 0, 1, 2, …, 9). Further details of this statistical approach are provided in our previous studies^16,18,36–40^.

**Phase 3***:* Optimizing the initial PSGD (*g* = 0) and estimating the threshold value using the GA algorithm in order to improve the predictive performance^39^. Specifically, the fitness function of the GA was mainly used for optimizing two important factors: the area under the receiver operating characteristic (AUC) (${W}_{1}$) and the Pearson’s correlation coefficient (R value) between the initial and optimized PSGD (*g* = 0) (${W}_{2}$). To avoid the overfitting issue, the fitness function $\mathrm{Fit}\left(.\right)$ was performed via a tenfold cross-validation procedure and represented as follows:

3 $$\mathrm{Fit}\left(\mathrm{PSGD}\right)=0.9\times \mathrm{AUC}+ 0.1\times \mathrm{R}$$

Furthermore, weights for ${W}_{1}$ and ${W}_{2}$ were set based on our previous studies^18,37–40^.

**Phase 4***:* Constructing a scoring function *S(P)* based on the SCM method to calculate TPP score of an unknown protein *P*. Herein, the scoring function was created using the optimized propensity scores of 400 dipeptides and can be defined as follows:

4 $$S(P)=\sum_{i=1}^{400}{DP}_{i}{PS}_{i}$$

where ${DP}_{i}$ and ${PS}_{i}$ represent the total number and propensity score of the *i*th dipeptide.

**Phase 5***:* Identifying the biological function of an unknown protein *P* using the scoring function S(*P*). Particularly, for a given unknown protein sequence *P*, it is classified as TPP if S(*P*) is greater than the threshold value, otherwise *P* is classified as non-TPP.

5 $$S\left(P\right)=\left\{\begin{array}{c}1,\sum_{i=1}^{400}{DP}_{i}{PS}_{i}>threshold\\\\0,\sum_{i=1}^{400}{DP}_{i}{PS}_{i}<threshold\end{array}\right.$$

where $1$ and $0$ represent prediction results as TPP and non-TPPs, respectively.

### Characterization of thermophilic proteins using SCMTPP

Propensity scores of 20 amino acids were estimated and used in this study to provide a better understanding of the biophysical and biochemical properties of TPPs using SCMTPP. Particularly, a statistical approach was used to calculate the propensity scores for each amino acid. The propensity score for Glu, for example, is calculated by averaging propensity scores of 40 dipeptides that contain Glu. In addition, propensity scores of 20 amino acids were also used to identify a set of informative physicochemical properties (PCPs) as extracted from the amino acid index database (AAindex)^41^ by means of R values from amongst propensity scores of 20 amino acids with those of 531 PCPs.

### Performance evaluation

In order to evaluate the prediction ability of the model, we used four widely used metrics for the two-class prediction problems as follows:

6 $$\mathrm{ACC}=\frac{\mathrm{TP}+\mathrm{TN}}{\left(\mathrm{TP}+\mathrm{TN}+\mathrm{FP}+\mathrm{FN}\right)}$$

7 $$\mathrm{Sn}=\frac{\mathrm{TP}}{\left(\mathrm{TP}+\mathrm{FN}\right)}$$

8 $$\mathrm{Sp}=\frac{\mathrm{TN}}{\left(\mathrm{TN}+\mathrm{FP}\right)}$$

9 $$\mathrm{MCC}=\frac{\mathrm{TP}\times \mathrm{TN}-\mathrm{FP}\times \mathrm{FN}}{\sqrt{(\mathrm{TP}+\mathrm{FP})(\mathrm{TP}+\mathrm{FN})(\mathrm{TN}+\mathrm{FP})(\mathrm{TN}+\mathrm{FN})}}$$

where ACC, Sn, Sp and MCC represents accuracy, sensitivity, specificity and Matthews correlation coefficient, respectively. Particularly, the number of correctly predicted true TPPs and true non-TPPs is indicated by TP and TN, respectively. Furthermore, FP stands for the number of non-TPPs that are predicted to be TPPs, and FN stands for the number of TPPs that was predicted to be non-TPPs. The proposed model was compared to previously described models using the receiver operating characteristic (ROC) curve of threshold-independent parameters. As a result, the area under the ROC curve (AUC) was used to evaluate prediction performance, with AUC values in the range of 0.5 and 1 denoting random and perfect models, respectively^42–47^.

### Analysis of three-dimensional structure of thermophilic proteins

Herein, Galaxy TBM (http://galaxy.seoklab.org/ index.html) was used for the determination of three-dimensional structures of TPPs and non-TPPs. The workflow of protein modelling consisted of two main stages: (i) selecting reliable models that are aligned with PROMALS3D^48^ and MODELLERCSA^49^ models and (ii) detecting and remodelling loop areas using the refining method. Particularly, protein structures of selected models were refined using 3Dpro (http://scratch.proteomics.ics.uci.edu/explanation.html#3Dpro) and GalaxyRefine (http://galaxy.seoklab.org/cgi-bin/submit.cgi?type = REFINE). Finally, the ProSA-web server (https://prosa.services.came.sbg.ac.at/prosa.php) and the Ramachandran plots were used to validate the three-dimensional structure. Moreover, hydrophobic and charge surface were visualized by using the BIOVIA Discovery Studio software (Dassault Systèmes BIOVIA, Discovery Studio Modeling Environment, Release 2018, San Diego: Dassault Systèmes, 2016).

## Results and discussion

### Prediction assessment of different propensity scores of g-gap dipeptides

The predictive performance of SCM classifiers trained with different PSGD (*g* = 0–9) was evaluated by means of tenfold cross-validation and independent tests on TPP-TRN and TPP-IND datasets, respectively. The GA algorithm was used to optimize and generate 10 sets of propensity scores for each g-gap dipeptide in order to construct 10 different SCM classifiers. As a result, among these ten sets, the one with the highest cross-validation MCC was chosen as the best. Supplementary Tables S1-S10 list the predictive performance of various SCM classifiers trained with PSGD (*g* = 0–9). Moreover, a summary of the predictive performance of 10 SCM classifiers trained by the 10 optimal sets of PSGD (*g* = 0–9) and evaluated by tenfold cross-validation and independent test results are recorded in Tables 2 and 3, respectively.

**Table 2:** Cross-validation results of SCM models using different optimal propensity scores of *g*-gap dipeptides.

*g*-gap	R	Cutoff	ACC	Sn	Sp	MCC	AUC
0	0.650	418	0.883	0.878	0.887	0.766	0.926
1	0.592	420	0.872	0.879	0.865	0.744	0.918
2	0.634	414	0.867	0.865	0.868	0.734	0.919
3	0.653	412	0.869	0.864	0.874	0.739	0.916
4	0.602	417	0.865	0.867	0.862	0.730	0.918
5	0.601	416	0.867	0.873	0.861	0.735	0.918
6	0.601	407	0.865	0.862	0.868	0.730	0.913
7	0.664	415	0.862	0.885	0.840	0.726	0.911
8	0.668	415	0.862	0.848	0.875	0.724	0.912
9	0.585	425	0.861	0.885	0.837	0.724	0.909
Mean	0.625	416	0.867	0.871	0.864	0.735	0.916
SD	0.032	4.77	0.006	0.012	0.015	0.013	0.005

**Table 3:** Independent test results of SCM models using different optimal propensity scores of *g*-gap dipeptides.

*g*-gap	R	Cutoff	ACC	Sn	Sp	MCC	AUC
0	0.650	418	0.865	0.849	0.881	0.731	0.925
1	0.592	420	0.844	0.846	0.841	0.687	0.912
2	0.634	414	0.863	0.868	0.857	0.725	0.918
3	0.653	412	0.860	0.836	0.884	0.721	0.908
4	0.602	417	0.852	0.863	0.841	0.704	0.909
5	0.601	416	0.852	0.854	0.849	0.704	0.915
6	0.601	407	0.867	0.863	0.871	0.733	0.914
7	0.664	415	0.853	0.860	0.846	0.706	0.909
8	0.668	415	0.840	0.822	0.857	0.680	0.910
9	0.585	425	0.837	0.849	0.825	0.674	0.897
Mean	416	0.625	0.853	0.851	0.855	0.706	0.912
SD	0.032	4.77	0.011	0.014	0.019	0.021	0.007

**Figure 2:**
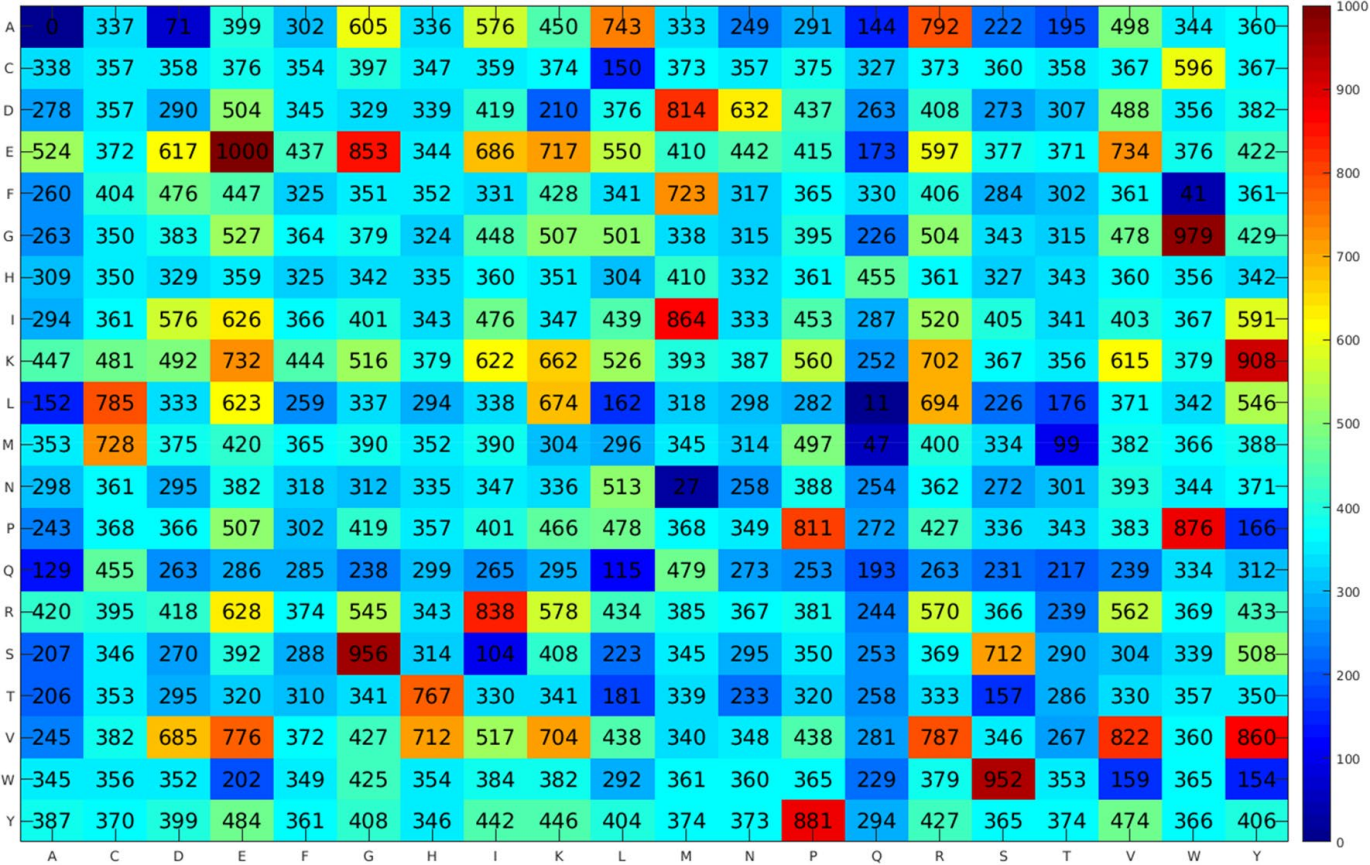
Propensity scores of 400 dipeptides as obtained from the proposed SCMTPP.

It is noticed that the mean ± SD values of ACC, Sn, Sp, MCC and AUC as based on 10 SCM classifiers are 0.867 ± 0.006, 0.871 ± 0.012, 0.864 ± 0.015, 0.735 ± 0.013 and 0.916 ± 0.005, respectively, using tenfold cross-validation. As can be seen from Table 2, PSGD (*g* = 0) was found to achieve the highest ACC of 0.883 with an MCC of 0.766 and an AUC of 0.926. Furthermore, PSGD (*g* = 1) and PSGD (*g* = 3) also performed well as it afforded the second and third highest ACC of 0.872 and 0.869, respectively. In the case of independent test results, Table 3 shows that the mean ± SD values of ACC, Sn, Sp, MCC and AUC based on 10 SCM classifiers are 0.850 ± 0.010, 0.842 ± 0.017, 0.858 ± 0.016, 0.700 ± 0.019 and 0.909 ± 0.006, respectively. PSGD (*g* = 6) achieved the highest ACC and MCC of 0.867 and 0.733, respectively, while PSGD (*g* = 0) achieved the second highest ACC and MCC of 0.865 and 0.731, respectively. From Table 3, it can be observed that PSGD (*g* = 0) achieved very comparable independent test results to that of PSGD (*g* = 6) in terms of all metrics (i.e. ACC, Sn, Sp, MCC and AUC). Taken into consideration the performance of both tenfold cross-validation and independent test results, results indicated that the SCM classifier trained with PSGD (*g* = 0) (i.e. the propensity scores of dipeptide) was the optimal one for the identification of TPPs and is referred to as SCMTPP. Further details of propensity scores of dipeptides are depicted in Fig. 2.

### Comparison of initial and optimized propensity scores

**Figure 3:**
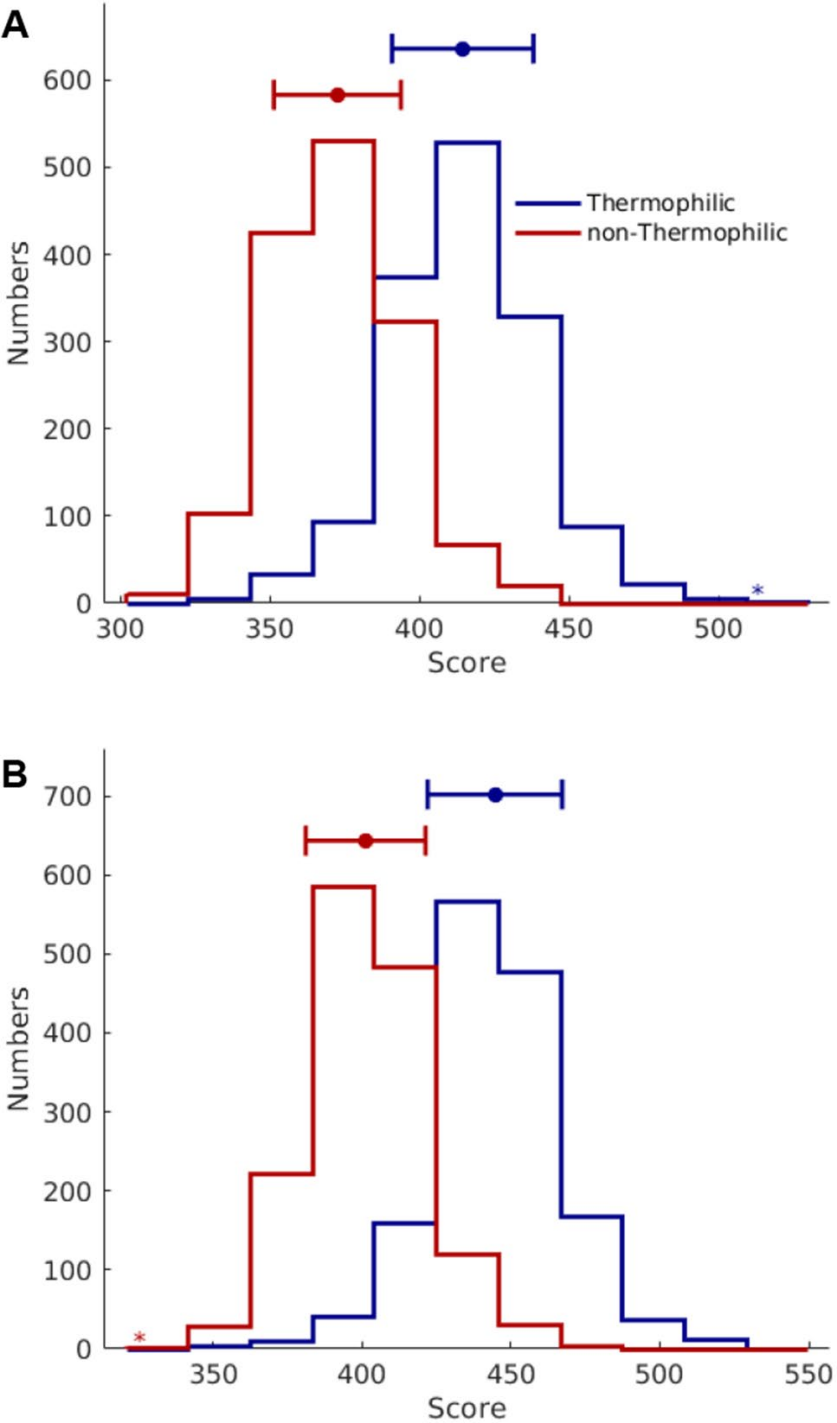
Histogram plot represent scores of thermophilic and non-thermophilic proteins as derived from SCMTPP using initial (**A**) and optimized (**B**) dipeptides propensity scores on the training dataset where the mean and standard deviation are indicated by bars and closed circles, respectively.

The improved predictive performance of SCMTPP is mainly due to estimated propensity scores of dipeptides derived from the SCM approach. In order to understand this phenomenon, firstly, we compared the predictive performance of optimized (optimized-PS) and initial (initial-PS) propensity scores of dipeptides. Table 4 shows the predictive performance of optimized-PS and initial-PS as evaluated by tenfold cross-validation and independent tests. As shown in Table 4, the optimized-PS achieved cross-validation ACC, Sp and MCC of 0.883, 0.887 and 0.766, which represents 3.9%, 5.8% and 7.8%, respectively, improvements over that of the initial-PS. Furthermore, independent test results of the optimized-PS were found to be consistently higher than that of the initial-PS. Particularly, optimized-PS afforded improvements as demonstrated by higher values of ACC, Sp and MCC of 1.7%, 3.7% and 3.8%, respectively, when compared to that of the initial-PS. In addition, histogram plots was used to represent scores of TTPs and non-TTPs as derived from SCMTPP by using initial-PS (Fig. 3A) and optimized-PS (Fig. 3B). As can be seen in Fig. 3, the optimized-PS shows a clear distinction between TTPs and non-TPPs thereby indicating that the optimized-PS was more effective for discriminating TTPs from non-TPPs than that of the initial-PS.

**Table 4:** Cross-validation and independent test results of SCM-based classifiers using initial-PS and optimized-PS.

Cross-validation	Feature	ACC	Sn	Sp	MCC	AUC
Tenfold CV	Initial-PS	0.844	0.858	0.829	0.688	0.910
	optimized-PS	0.883	0.878	0.887	0.766	0.926
Independent test	Initial-PS	0.848	0.852	0.844	0.695	0.914
	optimized-PS	0.865	0.849	0.881	0.731	0.925

### Comparison of SCMTPP with well-known ML classifiers and the existing method

**Figure 4:**
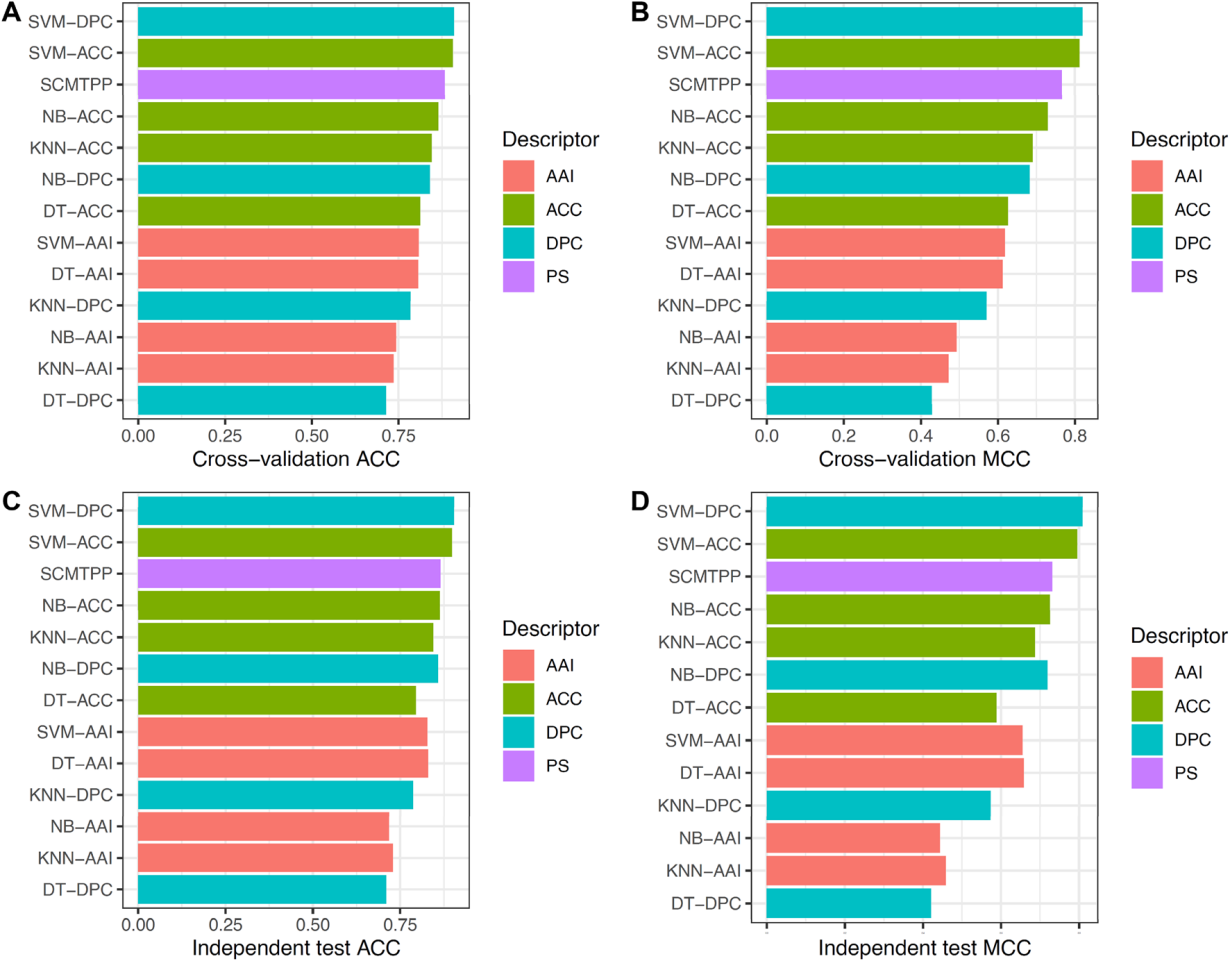
Performance evaluations of SCMTPP and conventional TPP predictors. (**A**,**B**) tenfold cross-validation of ACC and MCC from SCMTPP versus conventional TPP predictors. (**C**,**D**) Independent test of ACC and MCC from SCMTPP versus conventional TPP predictors.

In order to assess the predictive effectiveness of the proposed SCMTPP, we compared its performance with well-known ML classifiers as well as with the existing method on the same training and independent test dataset. Herein, we constructed and optimized several ML classifiers using SVM, decision tree (DT), k-nearest neighbor (KNN) and naive Bayes (NB) with AAC, DPC and amino acid index (AAI). All of these ML classifiers were constructed using the *scikit-learn* Python machine learning package (version 0.22)^50^. Figure 4 and Supplementary Tables S11-S12 summarize results of SCMTPP and several ML classifiers as evaluated by tenfold cross-validation and independent test. In regards to the existing method, Table 1 shows that three of these existing methods (i.e. Montanucci et al.’s method^21^, ThermoPred^20^ and Zuo et al.’s method^33^) were available as a webserver. However, ThermoPred is the only webserver that was functional at the time of this manuscript’s preparation. Therefore, the performance of SCMTPP was compared with only ThermoPred and their results are reported in Table 5.

**Table 5:** Cross-validation and independent test results of SCMTPP and ThermoPred.

Cross-validation	Method^a^	Ac	Sn	Sp	MCC
Tenfold CV	ThermoPred	–	–	–	–
	SCMTPP	0.883	0.878	0.887	0.766
Independent test	ThermoPred	0.860	0.938	0.782	0.729
	SCMTPP	0.865	0.849	0.881	0.731

^a^Results were obtained by feeding the protein sequences in the independent validation set to the web servers of ThermoPred.

Insights gained from Fig. 4, Table 5 and Supplementary Tables S11-S12 can be summarized as follows: (i) Two SVM-based classifiers consisting of SVM-DPC and SVM-ACC was found to achieve the two highest performance with ACC (cross-validation and independent test) of (0.910 and 0.904) and (0.906 and 0.898) for SVM-DPC and SVM-ACC, respectively; (ii) SCMTPP achieved very comparable to these two classifiers as well as ThermoPred with cross-validation and independent test ACC of 0.883 and 0.865, respectively, (iii) SCMTPP and SVM-based classifier (except for SVM-AAI) performed better than DT-based, KNN-based and NB-based classifiers. Particularly, the cross-validation ACC of SCMTPP was 7.05–16.83%, 3.78–14.68 and 1.86–14% higher than DT-based, KNN-based and NB-based classifiers, respectively. It is well-known that SVM method is a complicated approach that is not straightforward to provide the underlying biological implications^16,18,36–40^. On the other hand, SCM method is based on a simple weighted-sum approach that is more easy-to-understand method for biologists and provide interpretable propensity scores of dipeptides. Altogether, these comparative results revealed that the proposed SCMTPP predictor was the most suitable one for the identification and analysis of TPPs in terms of conceptual simplicity, ease of implementation and effectiveness.

### Identification of potential thermophilic proteins

**Figure 5:**
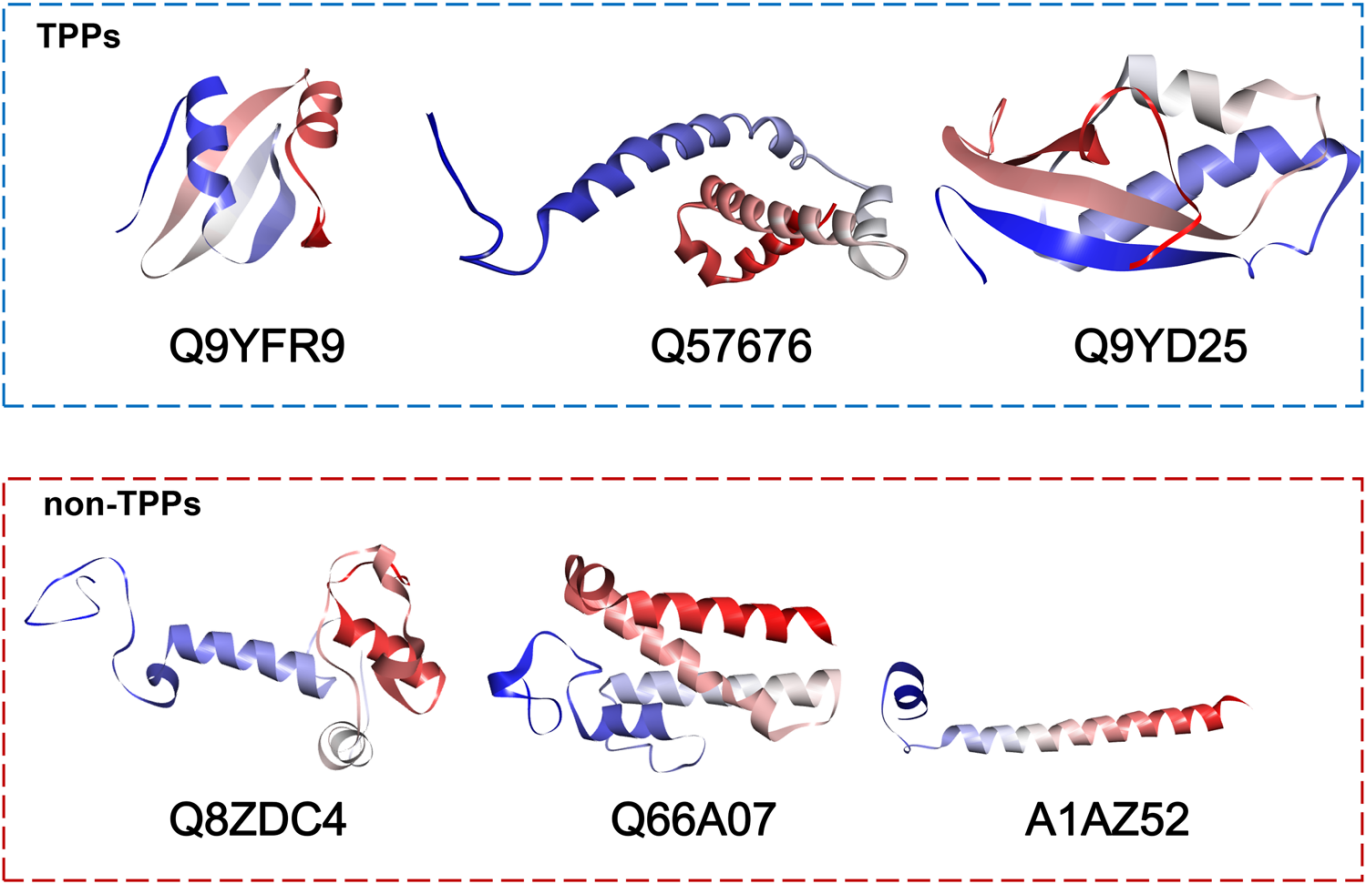
Three-dimensional structures of TPPs (Q9YFR9, Q57676 and Q9YD25) and non-TPPs (Q8ZDC4, Q66A07 and A1AZ52) having the highest (528.74, 527.79 and 525.29, respectively) and lowest (319.67, 331.20 and 340.61, respectively) TPP scores, respectively, where the optimal threshold value is 418.

Unlike existing methods, the proposed SCMTPP predictor is an easy-to-use and cost-effective for determining the likelihood of uncharacterized proteins namely TPPs using a simple scoring function $S(P)$^16,18,36–40^. Recently, Charoenkwan et al. made the use of SCM method for determining a new potential peptide-based drug for the hypoxia inducible factor 1α (HIF-1α)^36^. Herein, the scoring function $S\left(P\right)$ was used to calculate TPP scores (PS-TPP) for all proteins in the TPP-TRN dataset. Table 6 records ten top-ranked proteins having the highest TPP scores along with their name, PS-TPP, UniProt ID, function and source organism. As seen in Table Table 6, it could be noticed that all of the ten top-ranked proteins exhibited TPP scores of greater than 418. In addition, Fig. 5 depicts three-dimensional structures of TPPs (Q9YFR9, Q57676 and Q9YD25) and non-TPPs (Q8ZDC4, Q66A07 and A1AZ52) having the highest (528.74, 527.79 and 525.29, respectively) and lowest (319.67, 331.20 and 340.61, respectively) TPP scores, respectively. The five top-ranked proteins having the highest TPP scores and their UniProtID contained: 50S ribosomal protein L38E (528.74, Q9YFR9), Uncharacterized protein MJ0223 (527.79, Q57676), 50S ribosomal protein L31e (525.29, Q9YD25), Protein Grp (519.54, Q9WZV) and Elongation factor 1-beta (519.28, Q8TYN8). From amongst these ten proteins, they were from five main organisms consisting of *Aeropyrum pernix* (Q9YFR9, Q9YD25, P58289,), *Archaeoglobus fulgidus* (O28071), *Methanocaldococcus jannaschii* (Q57676), *Methanopyrus kandleri* (Q8TYN8, Q8TX34, Q8TXI4 and Q8TWL9) and *Thermotoga maritime (*Q9WZV4). Interestingly, the uncharacterized protein MJ0223 was from *Methanocaldococcus jannaschii* which is an anaerobic thermophilic archaea^51^.

**Table 6:** Top ten TPPs having the highest PS-TPP derived from the proposed SCMTPP.

Rank	Name (Uniprot)	PS-TPP	UniProt ID	Function	Organism
1	50S ribosomal protein L38E	528.74	Q9YFR9	Structural constituent of ribosome	*Aeropyrum pernix*
2	Uncharacterized protein MJ0223	527.79	Q57676	Unknown	*Methanocaldococcus jannaschii*
3	50S ribosomal protein L31e	525.29	Q9YD25	Structural constituent of ribosome	*Aeropyrum pernix*
4	Protein GrpE	519.54	Q9WZV4	Hyperosmotic and heat shock by preventing the aggregation of stress-denatured proteins	*Thermotoga maritima*
5	Elongation factor 1-beta	519.28	Q8TYN8	Promote the exchange of GDP for GTP in EF-1-alpha/GDP	*Methanopyrus kandleri*
6	50S ribosomal protein L29	518.45	Q8TX34	Structural constituent of ribosome	*Methanopyrus kandleri*
7	DNA double-strand break repair Rad50 ATPase	516.88	Q8TXI4	Facilitate opening of the processed DNA ends to aid in the recruitment of HerA and NurA	*Methanopyrus kandleri*
8	Putative antitoxin VapB21	516.77	O28071	Possibly the antitoxin component of a type II toxin-antitoxin (TA) system	*Archaeoglobus fulgidus*
9	V-type ATP synthase subunit E	514.51	Q8TWL9	Produces ATP from ADP in the presence of a proton gradient across the membrane	*Methanopyrus kandleri*
10	50S ribosomal protein L18Ae	513.46	P58289	Structural constituent of ribosome	*Aeropyrum pernix*

### Characterization of thermophilic proteins using propensity scores of amino acids

In this section, propensity scores of 20 amino acids and 400 dipeptides to be TPPs were analyzed to provide good understanding of physicochemical properties of TPP. As mentioned above, these propensity scores were generated by using SCMTPP based on the training dataset containing 1482 TPPs and 1482 non-TPPs. Table 7 records the propensity scores of amino acids along with the percentage of amino acid compositions, while Fig. 2 displays the propensity scores of dipeptides. As seen in Table 7, we notice that the correlation coefficient R between the propensity scores of amino acids and the difference of the percentage of amino acid compositions among TPPs and non-TPPs is 0.96. This again confirmed that the propensity scores of amino acids and dipeptides had more discriminative power to capture the key information between TPPs and non-TPPs. By consideration of the propensity scores of amino acids, we noticed that the top-five amino acids to be TPPs consisted of Glu, Lys, Val, Arg and Ile with respective scores of 510.18, 480.00, 470.75, 464.08 and 435.65, respectively, while the top-five amino acids to be non-TPPs consisted of Gln, Thr, Ala, Asn and Phe with respective scores of 255.43, 306.00, 323.63, 332.48 and 351.25, respectively. In case of the propensity scores of dipeptides, it could be found that the ten top-ranked dipeptides to be TPPs consisted of EE, GW, SG, WS, KY, YP, PW, IM, VY, EG and RI with their scores of 1000, 979, 956, 952, 908, 881, 876, 864, 860, 853 and 838, respectively, while the ten top-ranked dipeptides to be non-TPPs consisted of AA, LQ, NM, FW, MQ, AD, MT, SI, QL, QA and AQ with their scores of 0, 11, 27, 41, 47, 71, 99, 104, 115, 129 and 144, respectively.

**Table 7:** Propensity scores of twenty amino acids in becoming a thermophilic protein (PS-TPP) along with amino acid compositions (%) of TPPs and non-TPPs.

Amino acid	PS-TPP	TPP (%)	Non-TPP (%)	Difference
E-Glu	510.18 (1)	9.28	6.49	2.79 (1)
K-Lys	480.00 (2)	7.83	5.79	2.04 (2)
V-Val	470.75 (3)	8.45	7.09	1.36 (3)
R-Arg	464.08 (4)	6.47	5.14	1.32 (4)
I-Ile	435.65 (5)	7.41	6.45	0.96 (5)
G-Gly	433.48 (6)	7.34	7.12	0.22 (7)
Y-Tyr	425.93 (7)	3.42	2.89	0.53 (6)
P-Pro	421.40 (8)	4.26	4.13	0.13 (8)
C-Cys	388.28 (9)	0.92	1.07	− 0.15 (9)
M-Met	387.10 (10)	2.33	2.50	− 0.17 (11)
D-Asp	386.25 (11)	5.18	5.34	− 0.17 (10)
W-Trp	383.25 (12)	0.88	1.09	− 0.22 (12)
L-Leu	367.18 (13)	9.35	10.14	− 0.79 (15)
H-His	364.58 (14)	1.65	2.22	− 0.57 (14)
S-Ser	363.20 (15)	4.85	5.90	− 1.05 (17)
F-Phe	351.25 (16)	3.63	4.06	− 0.43 (13)
N-Asn	332.48 (17)	3.33	4.14	− 0.80 (16)
A-Ala	323.63 (18)	7.29	8.90	− 1.61 (19)
T-Thr	306.00 (19)	4.13	5.32	− 1.20 (18)
Q-Gln	255.43 (20)	2.01	4.21	− 2.20 (20)
R	1.00	0.54	0.12	0.96

As shown in Table 7, the ranks of the top-five amino acids to be TPPs (propensity, difference) for Glu, Lys, Val, Arg and Ile are (1, 1), (2, 2), (3, 3), (4, 4) and (5, 5), respectively, while the ranks of the top-five amino acids to be non-TPPs for Gln, Thr, Ala, Asn and Phe are (20, 20), (19, 18), (18, 19), (17, 16) and (16, 13), respectively. Many previous studies indicated that Glu, Lys and Arg had higher occurrence in TPPs than MPPs^20,27,28,35,52–55^. For example, Haney et al.^53^ conducted a comprehensive analysis on 115 protein sequences from *M. jannaschii.* Their results of amino acid composition analysis showed that Ile, Arg, Glu, Lys and Pro plays an important role in thermostability of proteins while Ser, Asn, Gln, Thr, and Met contributed to the mesostability of proteins. Haney et al.^53^ also reported that important physicochemical and biochemical properties for TPPs consisted of hydrophobicity, charged and uncharged polar residues. Zhang and Fang^35^ provided the residue distribution analysis by employing DPC on 3521 TPPs and 4895 MPP. Based on their analysis results, they reported that dipeptide compositions of EX and KX were significantly higher in TPPs as compared to MPPs while the dipeptide compositions of AX, HX, NX, QX and TX were significantly higher in MPPs as compared to TPPs where X denotes any amino acid. In 2004, Ding et al.^54^ mainly focused on the influence of single amino acid composition on TTPs by analyzing a large dataset containing three thermophilic organisms, ten hyperthermophilic organisms and 52 mesophilic organisms, which were collected from the NCBI database. From amongst 400 dipeptides, archaeal proteins had compositions of VK, KI, YK, IK, KV, KY and EV that were effective contributing to the increase of TPPs while compositions of DA, AD, TD, DD, DT, HD, DH, DR and DG contributed to the increase of MPPs. In the meanwhile, bacterial proteins had compositions of KE, EE, EK, YE, VK, KV, KK, LK, EI, EV, RK, EF, KY, VE, KI, KG, EY, FK, KF, FE, KR, VY, MK, WK and WE that contributed to the increase of TPPs while compositions of WQ, AA, QA, MQ, AW, QW, QQ, RQ, QH, HQ, AD, AQ, WL, QL, HA and DA contributed to the increase of MPPs. Altogether, our estimated propensity scores of amino acids as derived from SCMTPP is quite consistent with those of previous studies^20,27,28,54–56^. However, there are other factors responsible for improving the thermal stability of proteins such as hydrogen bonds, hydrophobic interactions, electrostatic interactions, α-helix forming and the entropy of unfolding^55,57^. More details on characterization of the thermal stability of proteins will be described below.

### Characterization of thermophilic proteins using informative PCPs

Numerous studies have demonstrated that biochemical and biophysical properties such as side chain^56,58^ or beta-sheet propensity^22^ and side chain^56,58^ were essential for understanding the thermostability of proteins. As can be seen in Table 8, the three selected informative PCPs along with their corresponding R values as selected by SCMTPP consisted of FUKS010101 (R = 0.616), FUKS010101 (R = 0.523) and FUKS010109 (R = 0.307), respectively. In addition, the top-twenty informative PCPs having the highest and lowest R values are recorded in Supplementary Tables S13 and S14, respectively.

**Table 8:** Summary of four important physicochemical properties as determined by SCMTPP.

Amino acid	PS-TPP (Rank)	FUKS010101 (Rank)	FUKS010102 (Rank)	ZIMJ680101 (Rank)
E-Glu	510.18 (1)	16.56 (1)	12.93 (1)	0.65 (13)
K-Lys	480.00 (2)	12.98 (2)	10.20 (2)	1.6 (7)
V-Val	470.75 (3)	4.05 (10)	3.57 (13)	1.79 (6)
R-Arg	464.08 (4)	8.48 (3)	6.87 (5)	0.83 (12)
I-Ile	435.65 (5)	3.3 (13)	2.72 (15)	3.07 (1)
G-Gly	433.48 (6)	8.29 (4)	7.95 (4)	0.1 (18)
Y-Tyr	425.93 (7)	2.75 (15)	2.26 (16)	2.97 (2)
P-Pro	421.40 (8)	5.41 (6)	4.79 (11)	2.7 (4)
C-Cys	388.28 (9)	0.29 (20)	0.31 (20)	1.48 (8)
M-Met	387.10 (10)	1.71 (18)	1.87 (18)	1.4 (9)
D-Asp	386.25 (11)	7.05 (5)	8.57 (3)	0.64 (14)
W-Trp	383.25 (12)	0.67 (19)	0.54 (19)	0.31 (16)
L-Leu	367.18 (13)	5.06 (7)	4.43 (12)	2.52 (5)
H-His	364.58 (14)	1.74 (17)	2.80 (14)	1.1 (10)
S-Ser	363.20 (15)	4.27 (9)	5.41 (8)	0.14 (17)
F-Phe	351.25 (16)	2.32 (16)	1.92 (17)	2.75 (3)
N-Asn	332.48 (17)	3.89 (11)	5.50 (7)	0.09 (19)
A-Ala	323.63 (18)	4.47 (8)	6.77 (6)	0.83 (11)
T-Thr	306.00 (19)	3.83 (12)	5.36 (9)	0.54 (15)
Q-Gln	255.43 (20)	2.87 (14)	5.24 (10)	0 (20)
R	1.00	0.616	0.348	0.307

**Figure 6:**
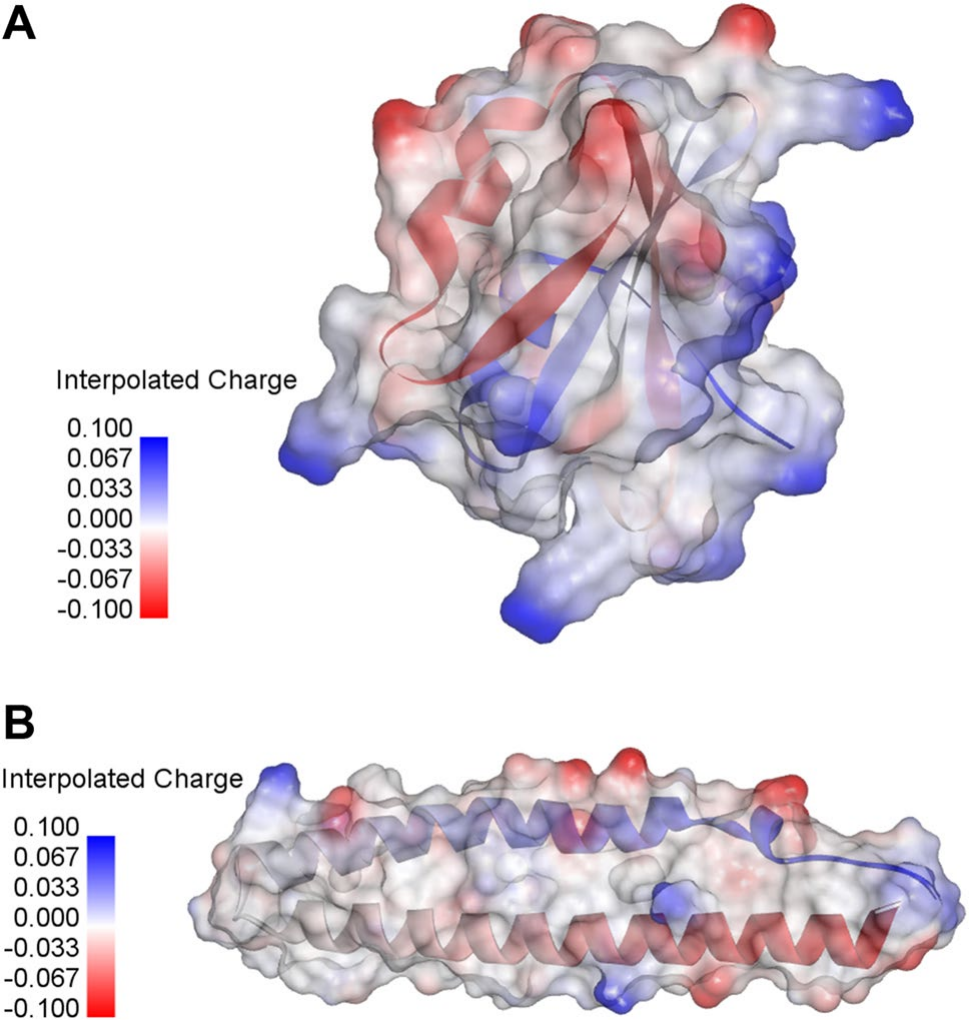
Interpolated charge surface of Q9YFR9 (TPP) and P0A223 (non-TPP) having TPP scores of 528.74 and 341.99, respectively, where the optimal threshold value is 418. Blue, white and red colors denote high, medium and low interpolated charge, respectively.

**Figure 7:**
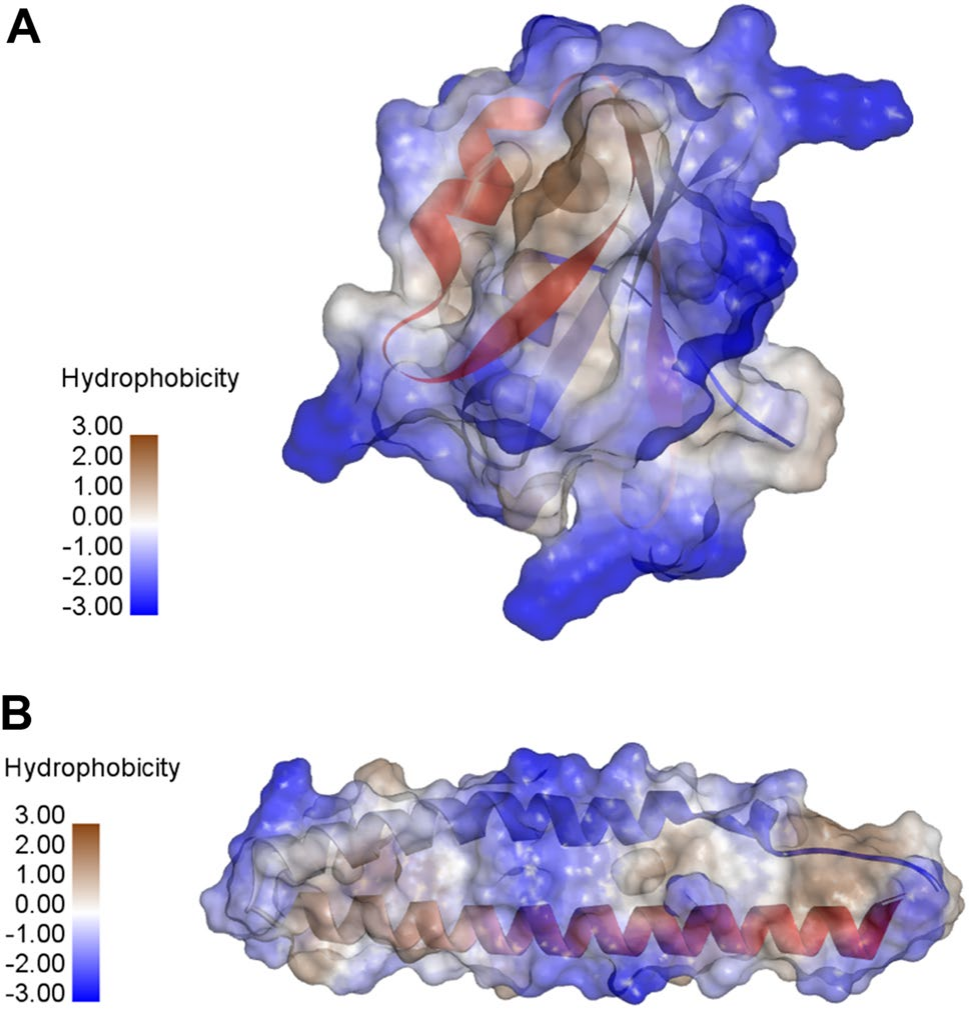
Surface hydrophobicity of Q9YFR9 (TPP) and P0A223 (non-TPP) having TPP scores of 528.74 and 341.99, respectively, where the optimal threshold value is 418. Brown, white and blue colors denote high, medium and low hydrophobicity, respectively.

The FUKS010101 property is described as the Surface composition of amino acids in intracellular proteins of thermophiles (percent) (Fukuchi-Nishikawa, 2001)^56^. Fukuchi and Nishikawa suggested that proteins from thermophilic bacteria had 45.1% charged residues containing 23.6% negatively charged residues and 21.5% positively charged residues on the surface, which was found to be higher than those of other groups (19.9% nonpolar residues, 16.6% polar residues and 18.5% others)^56^. Figure 6 provides an example on the interpolated charge surface plot of TPPs and non-TPPs. Figure 6A,B shows interpolated charge surface plots of Q9YFR9 (TPP) and P0A223 (non-TPP). The blue surfaces of the P0A223 indicates that the interpolated charge of the entire P0A223 is higher than that of P0A223. In general, the interpolated charge surface are often used to determine hydrogen bonding patterns, electrostatic interaction and strengths of salt bridges in biomolecular simulations^59^. Many studies have also confirmed that amino acids with charged side chains could be regarded as the important factor for the increase of the thermostability of proteins^35,57^ where positively and negatively charged amino acids contain (Arg, His and Lys) and (Asp and Glu), respectively. As shown in Table 8, the ranks of propensity scores (PS-TPP, FUKS010101) for Lys, Glu, Arg, Asp and His are (1, 1), (2, 2), (4, 3), (11, 5) and (14, 17), respectively. Interestingly, from amongst these charged amino acids, three of these were found in the top-five amino acids contributing to TPPs (i.e. Lys, Glu and Arg). At the typical biological pH, Lys and Glu is capable of carrying a charge for forming hydrogen bonds. This phenomenon render it as one of the crucial factors that is responsible for enhancing the thermostability of proteins. In the meanwhile, it is well-recognized that TTPs could participate in salt bridge interaction, which is known as a typical charge–charge interaction between oppositely charged residues. Many research groups have shown that the number of salt bridges show a positive correlation to the thermostability of proteins^35,60–63^. Interestingly, FUKS010101 and FUKS010102 properties are described in the AAindex as Surface composition of amino acids in intracellular proteins of thermophiles (percent) and mesophiles (percent) (Fukuchi-Nishikawa, 2001)^56^, respectively, while the ZIMJ680101 property is described in the AAindex as Hydrophobicity (Zimmerman et al., 1968). Specifically, FUKS010101 and FUKS010102 properties suggested that the fraction of hydrophobic residues in thermophilic bacteria (19.9%) is quite equivalent to that of the mesophilic bacteria (17.3%) in the surface composition^56^. Figure 7 shows an example surface hydrophobicity plot of TPPs and non-TPPs. Figure 7A,B shows surface hydrophobicity plots of Q9YFR9 (TPP) and P0A223 (non-TPP). Moreover, brown surfaces of Q9YFR9 was found to be quite similar to that of P0A223. Recently, Vieille and Zeikus^13^ conducted a comparative analysis of residue contents between TTPs and MPPs on genome sequences containing seven TTPs and eight MPPs. Their analysis revealed that the content of hydrophobic amino acids in TPPs was quite similar to those of MPPs. Vieille and Zeikus’s analysis were quite consistent with those of previous works^53,64,65^.

Herein, results from analyses were based on the propensity scores of 20 amino acids to be TPPs (i.e. derived from primary sequence information). Particularly, selected TPPs and non-TPPs were employed to analyze their interpolated charge and hydrophobicity. However, analysis was limited due to the small size of samples used herein. In order to explicitly understand this phenomenon, average values of interpolated charge and hydrophobicity from 1482 TPPs and 1482 non-TPPs should be computed for future analysis.

### Utilization of the proposed SCMTPP

Finally, we had created a user-friendly web server SCMTPP to allow easy access to the model by the scientific community. Thus, SCMTPP is freely available online at http://pmlabstack.pythonanywhere.com/SCMTPP. Step-by-step guidelines on how to use the SCMTPP web server are provided in the Supplementary information.

## Conclusions

The accurate identification of novel TTPs from a large number of uncharacterized protein sequences is important in basic research as well as a variety of applications in the food industry. Herein, we propose SCMTPP as a novel and interpretable computational model for the identification and characterization of TPPs. Firstly, we established an up-to-date dataset from published literature in order to develop an effective prediction model. Propensity scores of 20 amino acids and 400 g-gap dipeptides were calculated using the SCM method. Unlike previous methods, our predictor aims to provide a better understanding of the molecular basis for TPPs as well as improve prediction accuracy. Because of its simplicity, interpretability, and practical application, our empirical studies based on cross-validation and independent tests demonstrated the effectiveness and applicability of SCMTPP, which outperformed existing methods and widely used ML-based predictors. Finally, SCMTPP was set up as a publicly accessible web server at http://pmlabstack.pythonanywhere.com/SCMTPP to help experimental scientists with large-scale TPP identification. The proposed SCMTPP webserver and SCMTPP-derived propensity scores are expected to be useful tools for facilitating basic research and a variety of applications in the food industry.

## Supplementary Information

Supplementary Information.

## Data Availability

All the data are available at http://pmlabstack.pythonanywhere.com/SCMTPP.
