# Supplementary material for: A novel sequence-based predictor for identifying and characterizing thermophilic proteins using estimated propensity scores of dipeptides
Source: Sci Rep. 2021 Dec 10;11:23782. doi: 10.1038/s41598-021-03293-w (PMC8664844; doi:10.1038/s41598-021-03293-w)
Supplement: Supplementary file 1 — Supplementary Information. [file 41598_2021_3293_MOESM1_ESM.docx]

Step-by-step guidelines on how to use the SCMTPP web server

Firstly, the user opens the web server at <http://pmlabstack.pythonanywhere.com/SCMTPP> and the user will see the top screen of SCMTPP on the user’s computer screen. Secondly, the user enters the query sequence into the text box or uploads a FASTA file by clicking on the “Choose file” button. Thirdly, the user clicks on the *“*Submit” button in order to start the prediction process. Typically, in this step, it takes a few seconds for the server to process a task. Finally, after finishing the prediction process, the results are outputted as shown on the right-hand side of the web server. The user can see examples of FASTA-formatted sequences by clicking on the “example file” button.

**Table S1.** Cross-validation results of ten SCM classifiers with ten different propensity scores of 0-gap dipeptide (PSGD (g = 0))

| **#Exp** | **Threshold** | **Fitness**  **score** | **ACC** | **Sn** | **Sp** | **MCC** | **AUC** |
| --- | --- | --- | --- | --- | --- | --- | --- |
| 1 | 413 | 0.673 | 0.879 | 0.868 | 0.889 | 0.758 | 0.925 |
| 2 | 410 | 0.700 | 0.874 | 0.855 | 0.893 | 0.749 | 0.923 |
| 3 | 415 | 0.700 | 0.872 | 0.889 | 0.855 | 0.745 | 0.925 |
| 4 | 407 | 0.677 | 0.876 | 0.873 | 0.879 | 0.753 | 0.921 |
| **5** | 410 | 0.679 | 0.879 | 0.868 | 0.890 | 0.759 | 0.920 |
| 6 | 405 | 0.723 | 0.878 | 0.877 | 0.878 | 0.756 | 0.930 |
| 7 | 422 | 0.667 | 0.880 | 0.883 | 0.876 | 0.760 | 0.927 |
| **8** | **418** | **0.650** | **0.883** | **0.878** | **0.887** | **0.766** | **0.926** |
| 9 | 410 | 0.684 | 0.871 | 0.858 | 0.883 | 0.742 | 0.922 |
| 10 | 403 | 0.667 | 0.874 | 0.862 | 0.885 | 0.748 | 0.922 |
| Mean | 411.300 | 0.682 | 0.876 | 0.871 | 0.882 | 0.753 | 0.924 |
| STD. | 5.851 | 0.021 | 0.004 | 0.011 | 0.011 | 0.007 | 0.003 |

The experiment #8 having the highest cross-validation MCC is used for further analysis.

**Table S2.** Cross-validation results of ten SCM classifiers with ten different propensity scores of 1-gap dipeptide (PSGD (g = 1))

| **#Exp** | **Threshold** | **Fitness**  **score** | **ACC** | **Sn** | **Sp** | **MCC** | **AUC** |
| --- | --- | --- | --- | --- | --- | --- | --- |
| 1 | 433 | 0.583 | 0.866 | 0.860 | 0.872 | 0.732 | 0.925 |
| 2 | 407 | 0.586 | 0.863 | 0.866 | 0.860 | 0.727 | 0.912 |
| 3 | 415 | 0.548 | 0.865 | 0.860 | 0.870 | 0.731 | 0.915 |
| 4 | 412 | 0.616 | 0.868 | 0.860 | 0.877 | 0.737 | 0.919 |
| **5** | 409 | 0.607 | 0.869 | 0.851 | 0.887 | 0.739 | 0.918 |
| 6 | 417 | 0.585 | 0.859 | 0.858 | 0.859 | 0.718 | 0.910 |
| 7 | 411 | 0.582 | 0.863 | 0.844 | 0.881 | 0.727 | 0.917 |
| **8** | **420** | **0.592** | **0.872** | **0.879** | **0.865** | **0.744** | **0.918** |
| 9 | 417 | 0.612 | 0.862 | 0.842 | 0.882 | 0.725 | 0.912 |
| 10 | 411 | 0.603 | 0.858 | 0.843 | 0.873 | 0.717 | 0.912 |
| Mean | 415.200 | 0.591 | 0.864 | 0.856 | 0.873 | 0.730 | 0.916 |
| STD. | 7.436 | 0.020 | 0.004 | 0.012 | 0.009 | 0.009 | 0.005 |

The experiment #8 having the highest cross-validation MCC is used for further analysis.

**Table S3.** Cross-validation results of ten SCM classifiers with ten different propensity scores of 2-gap dipeptide (PSGD (g = 2))

| **#Exp** | **Threshold** | **Fitness**  **score** | **ACC** | **Sn** | **Sp** | **MCC** | **AUC** |
| --- | --- | --- | --- | --- | --- | --- | --- |
| 1 | 404 | 0.679 | 0.860 | 0.868 | 0.853 | 0.721 | 0.913 |
| 2 | 416 | 0.667 | 0.860 | 0.862 | 0.858 | 0.720 | 0.907 |
| 3 | 397 | 0.575 | 0.864 | 0.868 | 0.861 | 0.729 | 0.915 |
| 4 | 415 | 0.674 | 0.863 | 0.860 | 0.865 | 0.726 | 0.913 |
| 5 | 415 | 0.595 | 0.863 | 0.863 | 0.862 | 0.726 | 0.917 |
| 6 | 411 | 0.575 | 0.857 | 0.881 | 0.833 | 0.715 | 0.912 |
| 7 | 403 | 0.699 | 0.860 | 0.858 | 0.862 | 0.720 | 0.912 |
| **8** | **414** | **0.634** | **0.867** | **0.865** | **0.868** | **0.734** | **0.919** |
| 9 | 414 | 0.589 | 0.866 | 0.877 | 0.856 | 0.734 | 0.921 |
| 10 | 407 | 0.638 | 0.862 | 0.881 | 0.843 | 0.725 | 0.916 |
| Mean | 409.600 | 0.633 | 0.862 | 0.868 | 0.856 | 0.725 | 0.914 |
| STD. | 6.501 | 0.046 | 0.003 | 0.008 | 0.011 | 0.006 | 0.004 |

The experiment #8 having the highest cross-validation MCC is used for further analysis.

**Table S4.** Cross-validation results of ten SCM classifiers with ten different propensity scores of 3-gap dipeptide (PSGD (g = 3))

| **#Exp** | **Threshold** | **Fitness**  **score** | **ACC** | **Sn** | **Sp** | **MCC** | **AUC** |
| --- | --- | --- | --- | --- | --- | --- | --- |
| 1 | 417 | 0.660 | 0.862 | 0.855 | 0.869 | 0.724 | 0.914 |
| 2 | 417 | 0.575 | 0.862 | 0.872 | 0.853 | 0.725 | 0.916 |
| 3 | 429 | 0.534 | 0.866 | 0.845 | 0.887 | 0.733 | 0.917 |
| 4 | 418 | 0.573 | 0.864 | 0.853 | 0.876 | 0.729 | 0.916 |
| **5** | 419 | 0.620 | 0.862 | 0.854 | 0.871 | 0.725 | 0.914 |
| 6 | 415 | 0.654 | 0.866 | 0.858 | 0.875 | 0.733 | 0.920 |
| 7 | 429 | 0.598 | 0.863 | 0.862 | 0.864 | 0.726 | 0.915 |
| 8 | 422 | 0.583 | 0.867 | 0.860 | 0.874 | 0.734 | 0.924 |
| 9 | 425 | 0.624 | 0.866 | 0.883 | 0.850 | 0.734 | 0.920 |
| 10 | **412** | **0.653** | **0.869** | **0.864** | **0.874** | **0.739** | **0.916** |
| Mean | 420.300 | 0.607 | 0.865 | 0.861 | 0.869 | 0.730 | 0.917 |
| STD. | 5.794 | 0.042 | 0.002 | 0.011 | 0.011 | 0.005 | 0.003 |

The experiment #5 having the highest cross-validation MCC is used for further analysis.

**Table S5.** Cross-validation results of ten SCM classifiers with ten different propensity scores of 4-gap dipeptide (PSGD (g = 4))

| **#Exp** | **Threshold** | **Fitness**  **score** | **ACC** | **Sn** | **Sp** | **MCC** | **AUC** |
| --- | --- | --- | --- | --- | --- | --- | --- |
| 1 | 409 | 0.636 | 0.861 | 0.858 | 0.863 | 0.722 | 0.916 |
| 2 | 410 | 0.685 | 0.862 | 0.860 | 0.865 | 0.725 | 0.913 |
| 3 | 410 | 0.667 | 0.858 | 0.836 | 0.879 | 0.716 | 0.911 |
| **4** | **417** | **0.602** | **0.865** | **0.867** | **0.862** | **0.730** | **0.918** |
| 5 | 423 | 0.671 | 0.856 | 0.857 | 0.855 | 0.713 | 0.907 |
| 6 | 412 | 0.604 | 0.861 | 0.855 | 0.867 | 0.722 | 0.910 |
| 7 | 399 | 0.679 | 0.856 | 0.860 | 0.852 | 0.712 | 0.905 |
| 8 | 410 | 0.622 | 0.863 | 0.848 | 0.879 | 0.728 | 0.910 |
| 9 | 412 | 0.666 | 0.851 | 0.859 | 0.842 | 0.702 | 0.907 |
| 10 | 423 | 0.605 | 0.856 | 0.839 | 0.873 | 0.713 | 0.910 |
| Mean | 412.500 | 0.644 | 0.859 | 0.854 | 0.864 | 0.718 | 0.911 |
| STD. | 7.106 | 0.033 | 0.004 | 0.010 | 0.012 | 0.009 | 0.004 |

The experiment #4 having the highest cross-validation MCC is used for further analysis.

**Table S6.** Cross-validation results of ten SCM classifiers with ten different propensity scores of 5-gap dipeptide (PSGD (g = 5))

| **#Exp** | **Threshold** | **Fitness**  **score** | **ACC** | **Sn** | **Sp** | **MCC** | **AUC** |
| --- | --- | --- | --- | --- | --- | --- | --- |
| 1 | 414 | 0.616 | 0.857 | 0.864 | 0.849 | 0.714 | 0.907 |
| 2 | 418 | 0.689 | 0.863 | 0.858 | 0.868 | 0.727 | 0.911 |
| **3** | **416** | **0.601** | **0.867** | **0.873** | **0.861** | **0.735** | **0.918** |
| 4 | 404 | 0.631 | 0.860 | 0.847 | 0.872 | 0.720 | 0.913 |
| 5 | 421 | 0.601 | 0.858 | 0.857 | 0.859 | 0.717 | 0.908 |
| 6 | 414 | 0.614 | 0.859 | 0.881 | 0.837 | 0.719 | 0.912 |
| 7 | 429 | 0.628 | 0.860 | 0.841 | 0.880 | 0.722 | 0.915 |
| 8 | 409 | 0.629 | 0.851 | 0.853 | 0.849 | 0.703 | 0.907 |
| 9 | 408 | 0.622 | 0.854 | 0.841 | 0.868 | 0.709 | 0.905 |
| 10 | 406 | 0.645 | 0.862 | 0.860 | 0.864 | 0.724 | 0.917 |
| Mean | 413.900 | 0.627 | 0.859 | 0.857 | 0.861 | 0.719 | 0.911 |
| STD. | 7.593 | 0.025 | 0.005 | 0.013 | 0.013 | 0.009 | 0.004 |

The experiment #3 having the highest cross-validation MCC is used for further analysis.

**Table S7.** Cross-validation results of ten SCM classifiers with ten different propensity scores of 6-gap dipeptide (PSGD (g = 6))

| **#Exp** | **Threshold** | **Fitness**  **score** | **ACC** | **Sn** | **Sp** | **MCC** | **AUC** |
| --- | --- | --- | --- | --- | --- | --- | --- |
| 1 | 416 | 0.634 | 0.859 | 0.850 | 0.868 | 0.718 | 0.915 |
| 2 | 411 | 0.632 | 0.852 | 0.858 | 0.846 | 0.704 | 0.902 |
| 3 | 408 | 0.684 | 0.863 | 0.861 | 0.866 | 0.727 | 0.912 |
| 4 | 416 | 0.677 | 0.858 | 0.846 | 0.870 | 0.717 | 0.910 |
| 5 | 396 | 0.754 | 0.856 | 0.837 | 0.876 | 0.713 | 0.907 |
| 6 | 422 | 0.625 | 0.861 | 0.871 | 0.852 | 0.723 | 0.915 |
| 7 | 410 | 0.737 | 0.858 | 0.864 | 0.852 | 0.716 | 0.910 |
| 8 | 414 | 0.638 | 0.857 | 0.845 | 0.869 | 0.715 | 0.905 |
| **9** | **407** | **0.601** | **0.865** | **0.862** | **0.868** | **0.730** | **0.913** |
| 10 | 415 | 0.572 | 0.864 | 0.862 | 0.865 | 0.728 | 0.914 |
| Mean | 411.500 | 0.655 | 0.859 | 0.855 | 0.863 | 0.719 | 0.910 |
| STD. | 7.028 | 0.058 | 0.004 | 0.011 | 0.010 | 0.008 | 0.004 |

The experiment #9 having the highest cross-validation MCC is used for further analysis.

**Table S8.** Cross-validation results of ten SCM classifiers with ten different propensity scores of 7-gap dipeptide (PSGD (g = 7))

| **#Exp** | **Threshold** | **Fitness**  **score** | **ACC** | **Sn** | **Sp** | **MCC** | **AUC** |
| --- | --- | --- | --- | --- | --- | --- | --- |
| 1 | 412 | 0.717 | 0.847 | 0.854 | 0.841 | 0.696 | 0.900 |
| 2 | 415 | 0.676 | 0.862 | 0.860 | 0.865 | 0.725 | 0.915 |
| 3 | 415 | 0.593 | 0.860 | 0.862 | 0.858 | 0.720 | 0.912 |
| 4 | 427 | 0.637 | 0.856 | 0.856 | 0.856 | 0.712 | 0.908 |
| 5 | 406 | 0.674 | 0.854 | 0.864 | 0.845 | 0.709 | 0.909 |
| 6 | 410 | 0.647 | 0.854 | 0.851 | 0.858 | 0.709 | 0.909 |
| 7 | 415 | 0.703 | 0.855 | 0.853 | 0.856 | 0.710 | 0.908 |
| 8 | 417 | 0.627 | 0.858 | 0.864 | 0.852 | 0.716 | 0.918 |
| 9 | 411 | 0.646 | 0.860 | 0.855 | 0.864 | 0.720 | 0.915 |
| **10** | **415** | **0.664** | **0.862** | **0.885** | **0.840** | **0.726** | **0.911** |
| Mean | 414.300 | 0.658 | 0.857 | 0.860 | 0.853 | 0.714 | 0.910 |
| STD. | 5.519 | 0.037 | 0.005 | 0.010 | 0.009 | 0.009 | 0.005 |

The experiment #10 having the highest cross-validation MCC is used for further analysis.

**Table S9.** Cross-validation results of ten SCM classifiers with ten different propensity scores of 8-gap dipeptide (PSGD (g = 8))

| **#Exp** | **Threshold** | **Fitness**  **score** | **ACC** | **Sn** | **Sp** | **MCC** | **AUC** |
| --- | --- | --- | --- | --- | --- | --- | --- |
| 1 | 406 | 0.723 | 0.851 | 0.840 | 0.862 | 0.703 | 0.900 |
| 2 | 394 | 0.626 | 0.851 | 0.853 | 0.849 | 0.702 | 0.901 |
| **3** | **415** | **0.668** | **0.862** | **0.848** | **0.875** | **0.724** | **0.912** |
| 4 | 413 | 0.672 | 0.857 | 0.856 | 0.859 | 0.715 | 0.908 |
| 5 | 396 | 0.622 | 0.860 | 0.865 | 0.856 | 0.721 | 0.906 |
| 6 | 407 | 0.641 | 0.860 | 0.860 | 0.860 | 0.721 | 0.910 |
| 7 | 401 | 0.737 | 0.851 | 0.831 | 0.871 | 0.703 | 0.903 |
| 8 | 417 | 0.618 | 0.860 | 0.860 | 0.860 | 0.720 | 0.912 |
| 9 | 413 | 0.665 | 0.856 | 0.843 | 0.870 | 0.713 | 0.901 |
| 10 | 399 | 0.596 | 0.856 | 0.858 | 0.853 | 0.711 | 0.909 |
| Mean | 406.100 | 0.657 | 0.856 | 0.851 | 0.861 | 0.713 | 0.906 |
| STD. | 8.293 | 0.046 | 0.004 | 0.011 | 0.008 | 0.008 | 0.005 |

The experiment #3 having the highest cross-validation MCC is used for further analysis.

**Table S10.** Cross-validation results of ten SCM classifiers with ten different propensity scores of 9-gap dipeptide (PSGD (g = 9))

| **#Exp** | **Threshold** | **Fitness**  **score** | **ACC** | **Sn** | **Sp** | **MCC** | **AUC** |
| --- | --- | --- | --- | --- | --- | --- | --- |
| 1 | 413 | 0.614 | 0.861 | 0.879 | 0.844 | 0.723 | 0.916 |
| **2** | **425** | **0.585** | **0.861** | **0.885** | **0.837** | **0.724** | **0.909** |
| 3 | 429 | 0.617 | 0.853 | 0.843 | 0.862 | 0.707 | 0.905 |
| 4 | 408 | 0.612 | 0.860 | 0.856 | 0.864 | 0.721 | 0.915 |
| 5 | 387 | 0.597 | 0.856 | 0.853 | 0.859 | 0.712 | 0.906 |
| 6 | 428 | 0.658 | 0.854 | 0.855 | 0.852 | 0.708 | 0.911 |
| 7 | 408 | 0.643 | 0.859 | 0.847 | 0.871 | 0.720 | 0.912 |
| 8 | 427 | 0.585 | 0.861 | 0.855 | 0.867 | 0.723 | 0.916 |
| 9 | 408 | 0.603 | 0.851 | 0.860 | 0.843 | 0.703 | 0.897 |
| 10 | 395 | 0.614 | 0.857 | 0.857 | 0.857 | 0.714 | 0.909 |
| Mean | 412.800 | 0.613 | 0.857 | 0.859 | 0.856 | 0.715 | 0.910 |
| STD. | 14.513 | 0.023 | 0.004 | 0.013 | 0.011 | 0.008 | 0.006 |

The experiment #2 having the highest cross-validation MCC is used for further analysis.

**Table S11.** Performance comparison of SCMTPP with conventional machine learning classifiers on the training dataset.

| **Method** | **ACC** | **Sn** | **Sp** | **MCC** | **AUC** |
| --- | --- | --- | --- | --- | --- |
| SVM-DPC | 0.910 | 0.920 | 0.899 | 0.820 | 0.968 |
| SVM-ACC | 0.906 | 0.906 | 0.906 | 0.812 | 0.965 |
| SCM | 0.883 | 0.878 | 0.887 | 0.766 | 0.926 |
| NB-ACC | 0.864 | 0.875 | 0.853 | 0.729 | 0.925 |
| KNN-ACC | 0.845 | 0.852 | 0.838 | 0.690 | 0.845 |
| NB-DPC | 0.840 | 0.868 | 0.812 | 0.682 | 0.911 |
| DT-ACC | 0.812 | 0.807 | 0.817 | 0.626 | 0.812 |
| SVM-AAI | 0.808 | 0.797 | 0.820 | 0.618 | 0.879 |
| DT-AAI | 0.806 | 0.801 | 0.811 | 0.612 | 0.806 |
| KNN-DPC | 0.784 | 0.832 | 0.736 | 0.570 | 0.784 |
| NB-AAI | 0.743 | 0.829 | 0.656 | 0.493 | 0.821 |
| KNN-AAI | 0.736 | 0.736 | 0.736 | 0.472 | 0.736 |
| DT-DPC | 0.714 | 0.712 | 0.717 | 0.429 | 0.714 |
| Mean | 0.819 | 0.832 | 0.807 | 0.640 | 0.853 |
| STD | 0.057 | 0.052 | 0.074 | 0.114 | 0.075 |

**Table S12.** Performance comparison of SCMTPP with conventional machine learning classifiers on the independent test dataset.

| **Method** | **ACC** | **Sn** | **Sp** | **MCC** | **AUC** |
| --- | --- | --- | --- | --- | --- |
| SVM-DPC | 0.904 | 0.906 | 0.903 | 0.809 | 0.969 |
| SVM-ACC | 0.898 | 0.911 | 0.884 | 0.795 | 0.962 |
| SCM | 0.865 | 0.849 | 0.881 | 0.731 | 0.925 |
| NB-ACC | 0.863 | 0.876 | 0.849 | 0.725 | 0.923 |
| NB-DPC | 0.858 | 0.900 | 0.817 | 0.719 | 0.912 |
| KNN-ACC | 0.844 | 0.844 | 0.844 | 0.687 | 0.844 |
| DT-AAI | 0.829 | 0.827 | 0.830 | 0.658 | 0.829 |
| SVM-AAI | 0.827 | 0.825 | 0.830 | 0.655 | 0.891 |
| DT-ACC | 0.794 | 0.790 | 0.798 | 0.588 | 0.794 |
| KNN-DPC | 0.786 | 0.819 | 0.752 | 0.573 | 0.786 |
| KNN-AAI | 0.729 | 0.706 | 0.752 | 0.459 | 0.729 |
| NB-AAI | 0.718 | 0.811 | 0.625 | 0.444 | 0.805 |
| DT-DPC | 0.710 | 0.693 | 0.728 | 0.421 | 0.710 |
| Mean | 0.817 | 0.827 | 0.807 | 0.636 | 0.852 |
| STD | 0.060 | 0.058 | 0.076 | 0.118 | 0.077 |

**Table S13.** The twenty top-ranked informative physicohemical properties having the highest pearson correlation (R) with the propensity scores of amino acids

| **Rank** | **AAindex** | **R** | **Description** |
| --- | --- | --- | --- |
| 1 | FUKS010101 | 0.616 | Surface composition of amino acids in intracellular proteins of thermophiles (percent) (Fukuchi-Nishikawa, 2001) |
| 2 | FUKS010109 | 0.523 | Entire chain composition of amino acids in intracellular proteins of thermophiles (percent) (Fukuchi-Nishikawa, 2001) |
| 3 | AURR980120 | 0.492 | Normalized positional residue frequency at helix termini C4' (Aurora-Rose, 1998) |
| 4 | AURR980101 | 0.491 | Normalized positional residue frequency at helix termini N4'(Aurora-Rose, 1998) |
| 5 | RACS820103 | 0.472 | Average relative fractional occurrence in AL(i) (Rackovsky-Scheraga, 1982) |
| 6 | ZIMJ680103 | 0.428 | Polarity (Zimmerman et al., 1968) |
| 7 | EISD860102 | 0.393 | Atom-based hydrophobic moment (Eisenberg-McLachlan, 1986) |
| 8 | FUKS010102 | 0.348 | Surface composition of amino acids in intracellular proteins of mesophiles (percent) (Fukuchi-Nishikawa, 2001) |
| 9 | FUKS010104 | 0.330 | Surface composition of amino acids in nuclear proteins (percent) (Fukuchi-Nishikawa, 2001) |
| 10 | HOPT810101 | 0.329 | Hydrophilicity value (Hopp-Woods, 1981) |
| 11 | HOPA770101 | 0.327 | Hydration number (Hopfinger, 1971), Cited by Charton-Charton (1982) |
| 12 | KUMS000101 | 0.314 | Distribution of amino acid residues in the 18 non-redundant families of thermophilic proteins (Kumar et al., 2000) |
| 13 | ZIMJ680101 | 0.307 | Hydrophobicity (Zimmerman et al., 1968) |
| 14 | DIGM050101 | 0.303 | Hydrostatic pressure asymmetry index, PAI (Di Giulio, 2005) |
| 15 | AURR980106 | 0.302 | Normalized positional residue frequency at helix termini N1 (Aurora-Rose, 1998) |
| 16 | ENGD860101 | 0.300 | Hydrophobicity index (Engelman et al., 1986) |
| 17 | FAUJ880112 | 0.299 | Negative charge (Fauchere et al., 1988) |
| 18 | PRAM900101 | 0.299 | Hydrophobicity (Prabhakaran, 1990) |
| 19 | LEVM760101 | 0.298 | Hydrophobic parameter (Levitt, 1976) |
| 20 | RICJ880104 | 0.297 | Relative preference value at N1 (Richardson-Richardson, 1988) |

**Table S14.** The twenty top-ranked informative physicohemical properties having the lowest pearson correlation (R) with the propensity scores of amino acids

| **Rank** | **AAindex** | **R** | **Description** |
| --- | --- | --- | --- |
| 1 | SNEP660104 | -0.491 | Principal component IV (Sneath, 1966) |
| 2 | ANDN920101 | -0.363 | alpha-CH chemical shifts (Andersen et al., 1992) |
| 3 | COSI940101 | -0.343 | Electron-ion interaction potential values (Cosic, 1994) |
| 4 | VELV850101 | -0.343 | Electron-ion interaction potential (Veljkovic et al., 1985) |
| 5 | BUNA790102 | -0.337 | alpha-CH chemical shifts (Bundi-Wuthrich, 1979) |
| 6 | QIAN880114 | -0.337 | Weights for beta-sheet at the window position of -6 (Qian-Sejnowski, 1988) |
| 7 | AURR980105 | -0.324 | Normalized positional residue frequency at helix termini Nc (Aurora-Rose, 1998) |
| 8 | NAGK730102 | -0.310 | Normalized frequency of bata-structure (Nagano, 1973) |
| 9 | SNEP660101 | -0.307 | Principal component I (Sneath, 1966) |
| 10 | NAKH900106 | -0.302 | Normalized composition from animal (Nakashima et al., 1990) |
| 11 | JACR890101 | -0.294 | Weights from the IFH scale (Jacobs-White, 1989) |
| 12 | YUTK870102 | -0.291 | Unfolding Gibbs energy in water, pH9.0 (Yutani et al., 1987) |
| 13 | CHOP780212 | -0.284 | Frequency of the 1st residue in turn (Chou-Fasman, 1978b) |
| 14 | WERD780102 | -0.278 | Free energy change of epsilon(i) to epsilon(ex) (Wertz-Scheraga, 1978) |
| 15 | NAKH900104 | -0.277 | Normalized composition of mt-proteins (Nakashima et al., 1990) |
| 16 | WERD780103 | -0.276 | Free energy change of alpha(Ri) to alpha(Rh) (Wertz-Scheraga, 1978) |
| 17 | RICJ880103 | -0.274 | Relative preference value at N-cap (Richardson-Richardson, 1988) |
| 18 | RICJ880109 | -0.266 | Relative preference value at Mid (Richardson-Richardson, 1988) |
| 19 | RICJ880114 | -0.262 | Relative preference value at C1 (Richardson-Richardson, 1988) |
| 20 | TANS770102 | -0.260 | Normalized frequency of isolated helix (Tanaka-Scheraga, 1977) |
